# Protocol deviation outlier estimation combined with generative AI

**DOI:** 10.1186/s13104-026-07823-5

**Published:** 2026-04-10

**Authors:** Ioannis I. Spyroglou, Magdaléna Řeháková, Radim Klapka, Anna Kacprzycka, Maria Consuelo Luguera Fernandez, Ronojit Adyanthaya, Kyle Young, Jolie Weintraub

**Affiliations:** Merck & Co., Inc., Rahway, NJ 07065 USA

**Keywords:** Protocol deviations, Clinical trials, Outlier detection, Generative AI, Risk management, Data quality

## Abstract

**Objective:**

Protocol deviations (PDs) are critical indicators of data quality and operational risk in clinical trials. Systematic under- or over-reporting of PDs at investigator sites can obscure true trial performance and compromise regulatory compliance. The goal of this paper is to present an integrated approach combining statistical outlier detection with generative AI-powered content analysis for detecting and characterizing outlier sites in PD reporting, enabling proactive risk management and targeted site oversight.

**Results:**

In this study, the simaerep bootstrap algorithm was applied across 578 clinical studies encompassing 39,936 sites. It is shown that the method successfully identifies sites exhibiting statistically significant deviations from expected PD reporting rates, flagging both potential under-reporters and high-rate reporters. In addition, generative AI combined with hierarchical clustering automatically categorized diverse PD narratives into a validated taxonomy of 15 major categories and 47 specific topics through a "human-in-the-loop" refinement process with clinical operations experts. Independent validation of the GenAI topic classification using an "LLM-as-judge" approach achieved 88.7% accuracy (95% CI: 86.5%–90.6%). This integrated capability equips clinical operations teams to identify sites warranting further investigation and determine whether observed deviation patterns are study-systemic, country-specific, or site-specific, ultimately improving data integrity and risk-based audit strategy.

## Introduction

Protocol deviations directly impact data integrity, participant safety, and regulatory compliance [1–4], making accurate identification and classification essential for trial quality [5]. Traditional monitoring relies on manual review, often failing to detect systematic patterns across sites [6], while deviation rates vary significantly due to training, resources, and local practices [7]. Recent advances in statistical methodology and AI enable proactive risk management [8–9]. The simaerep bootstrap algorithm identifies sites with atypical reporting patterns [10], and generative AI enables analysis of unstructured clinical narratives. Integrating quantitative outlier detection with qualitative content analysis enables comprehensive risk assessment and targeted interventions.

## Methodology

### Study design and data sources

This methodology was developed using clinical trial data from multiple therapeutic areas and geographic regions, consisting of two components: (1) statistical outlier detection using the bootstrap algorithm, and (2) AI-powered topic modeling of PD narratives.

### Protocol deviation outlier detection

The bootstrap algorithm (simaerep) was adapted from its original application in adverse event underreporting to identify investigator sites with atypical PD reporting patterns [10].

Operational Definition: Visits are scheduled protocol-specified participant encounters representing discrete time points where deviations may be recorded. Visit count per participant serves as the exposure denominator for site-level PD rates.The algorithm follows a six-step process:

Step-1: Record initial PDs per visits rate on site level.

Step-2: Replace every participant with a random participant from the same study with the same number of visits.

Step-3: Record PDs per visits rate for new set of participants.

Step-4: Repeat Steps 2, 3 a total of 1,000 times to generate a distribution of expected mean PD counts. The 1,000-iteration threshold balances computational efficiency with statistical precision. Convergence analysis demonstrated probability estimates stabilized (coefficient of variation(CV) < 1%) after approximately 800 iterations, aligning with bootstrap literature recommendations [10, 11].

Step-5: Calculate the probability of obtaining a mean cumulative PD count equal to or lower/higher than that observed in Step 1.

Step-6: Adjust probabilities using the Benjamini-Hochberg procedure to control false discovery rate (FDR) at α = 0.05. This approach maintains statistical power while controlling for multiplicity in multi-site evaluations.

The bootstrap resampling assumes participant exchangeability across sites conditional on visit count. While inter-site differences may introduce heterogeneity (despite randomization in randomized controlled trials (RCTs)), within-study resampling preserves protocol-specific context, and the method serves as a signal detection tool rather than a definitive classifier. To ensure reliable estimates, studies with ≤ 3 sites and sites with < 2 subjects are excluded; those falling below these thresholds are flagged for manual review. Synthetic datasets for exploring algorithm behavior are available in the simaerep repository (https://github.com/openpharma/simaerep).

We implemented GenAI topic extraction specific to each study, enabling topic distribution comparison at investigator sites. Study-level hierarchical topic merging approach using generative AI and clustering techniques was tested in order to evaluate future possibility for over-study (portfolio) topics comparison. GPT-4.1 was used for topic creation and text-embedding-ada-002-2 for embeddings [12, 13]. Other models can also be used with similar performance.

Prompt engineering followed a structured template approach ensuring consistent, parseable responses, with: (1) clear task specification, (2) contextual input presentation, and (3) structured output format requirements. Temperature was set to 0 to maximize reproducibility. The text-embedding-ada-002-2 model was used for semantic embedding generation, enabling cosine similarity-based clustering.

**Fig. 1:**
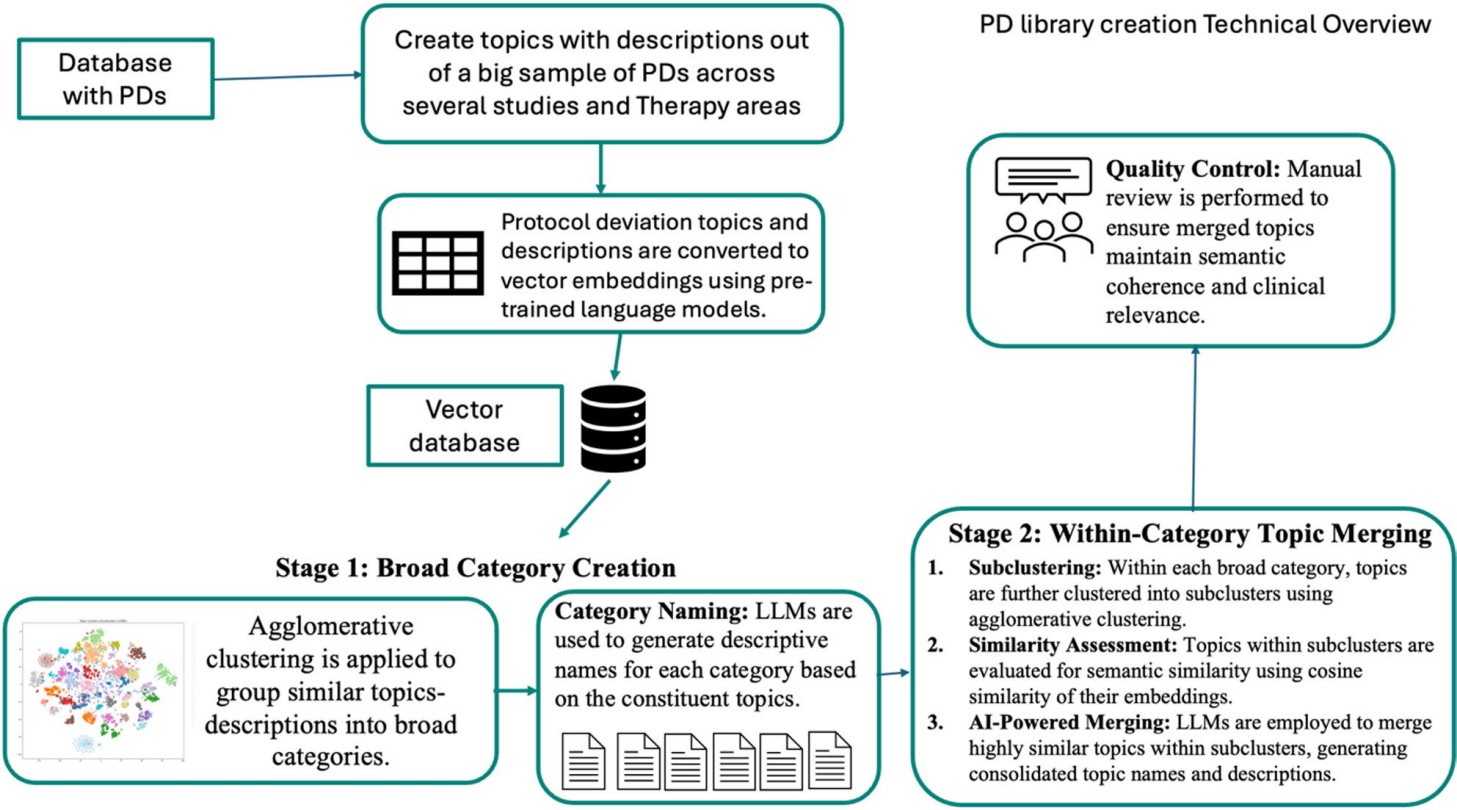
Technical workflow of the PD topics/categories library creation

The current implementation in Fig. 1 processes English-language narratives. For multilingual trials, narratives were documented in English or translated prior to analysis. Future implementations could incorporate multilingual embedding models and language detection preprocessing reducing potential translation-induced information loss.

### Integration and risk assessment

The outputs from both the statistical outlier detection and AI-powered topic analysis were integrated to provide comprehensive site risk profiles. Sites flagged as statistical outliers were cross-referenced with their deviation topic distributions to identify specific concerns and guide targeted interventions.

## Results

### Protocol deviation outlier detection results

Key patterns identified included:

- High-rate outliers: Sites with exceptionally high PD rates.
- Low-rate outliers: Sites with suspiciously low reporting rates, suggesting potential underreporting.
- Geographic clustering: Certain countries showed consistent patterns of specific deviation types.

### AI-powered topic analysis results

The GenAI topic-extraction approach successfully introduces a study-specific protocol-deviation taxonomy. Outlier detection, together with topic quantification, enables focused discussion of existing and potential challenges with protocol conduct at the site level. The hierarchical topic-merging approach successfully processes diverse protocol-deviation narratives, creating a structured taxonomy of deviation categories. The analysis revealed several themes:

- Sample Collection and Laboratory Issues
- Informed Consent and Documentation
- Visit Scheduling and Timing
- Medication Administration
- Adverse Event Reporting

### Quantitative performance assessment

The methodology was applied across 578 clinical studies encompassing 39,936 sites. Bootstrap analysis demonstrated stable convergence (CV < 1% after 800 iterations).

Probability thresholds are determined study-specifically, using median site-level probabilities as reference, ensuring sites are evaluated relative to study-specific context.

Following Benjamini-Hochberg adjustment, approximately 9% of sites were classified as potential under-reporters and 20% as high-rate reporters.

Hierarchical topic modeling reduced 6,284 initial topics to 358 across 40 broad categories (silhouette-score: 0.36, reasonable cluster separation). This broad output was intentionally configured at experts’ request to ensure comprehensive clinical coverage. Experts iteratively refined it to a validated taxonomy of 15 major categories and 47 topics by merging redundant topics, splitting overly broad categories, and standardizing terminology to align with clinical workflows. Limitations included inconsistent performance on rare deviation types (< 1% of total deviations).

- Classification accuracy in Table 1 was assessed using GPT-5.2 as an independent LLM-as-judge evaluator, following established practices in LLM evaluation literature [14, 15]. The judge LLM’s output was checked by a clinical expert.

**Table 1:** Classification validation results and error analysis

Sample size	1,000	Randomly selected PD narratives
Classification accuracy	88.7%	887/1,000 correctly classified
95% CI (Wilson Score)	86.5% - 90.6%	—

Error type	Proportion	Description
Boundary cases	~ 35%	Deviations spanning multiple categories
Ambiguous narratives	~ 25%	Abbreviated or unclear source text
Rare deviation types	~ 15%	Limited training representation
Straightforward errors	~ 15%	Incorrect topic selection

This accuracy demonstrates robust performance for automated classification of free-text clinical narratives with inherent variability in terminology and documentation styles.

### Impact on audit strategy and compliance

The methodology demonstrates significant value in real audit scenarios, leading to several important outcomes:

**Trend Identification**: The algorithm successfully identifies emerging patterns in PD reporting that were not immediately apparent through traditional monitoring approaches.

For site-level audits, topic trend analysis reduces time needed to track deviation reoccurrence and assess action item effectiveness. At the study level, it enables discovery of complex issues—for example, deviation proportions within topics may vary significantly across countries, enabling targeted resolution of region-specific issues and identification of underlying systemic root causes.

**Enhanced Compliance**: Focused remediation at flagged sites improves protocol adherence.

**Proactive Risk Management**: The early identification of outlier investigator sites enables proactive interventions before significant compliance issues developed, preventing potential regulatory concerns and data integrity problems.

**Resource Allocation**: Risk profiling concentrates oversight on high-risk sites.

Due to the confidential nature of clinical trial data, specific case examples cannot be disclosed. The methodology has been applied across multiple therapeutic areas and geographic regions, with findings used to inform risk-based monitoring strategies. Generalized application scenarios are provided to illustrate practical utility (see Table 2). Organizations implementing this methodology are encouraged to develop internal case libraries to support training and continuous improvement.

**Table 2:** Practical application scenarios

Scenario A: Identification of Under-Reporting Signals	The methodology identifies sites where the mean PD count is statistically lower than expected based on the distribution of other sites within the study. This signal prompts further investigation by auditors—it does not constitute a definitive finding of under-reporting but rather prioritizes sites for expert review.
Scenario B: Targeted Training Intervention	Sites flagged as high-rate outliers can be further characterized by their predominant deviation topics. This enables targeted intervention strategies—for example, sites with elevated “Informed Consent” deviations may benefit from consent process retraining, while sites with “Sample Collection” issues may require laboratory procedure refreshers.
Scenario C: Cross-Study Benchmarking	While probability thresholds are study-specific, the topic taxonomy generated through hierarchical merging provides a standardized framework for comparing deviation patterns across studies within the same therapeutic area, supporting organizational learning and protocol design optimization.

## Discussion

### Methodological advantages

Integrating statistical outlier detection with AI-powered content analysis advances clinical trial risk management. Outlier detection identifies that a site deviates but not why; topic analysis characterizes content but cannot determine statistical significance. The integrated approach enables actionable intelligence—for example, a high-rate outlier dominated by ‘Informed Consent’ deviations receives targeted consent intervention, while one with ‘Sample Collection’ issues receives laboratory retraining [10, 16–17]. Auditors receive complete topic distributions and historical action item data, enabling them to determine whether patterns are study-systemic, country-specific, or site-specific, and to track whether deviations reappear after corrective actions. The hierarchical topic modeling creates broad categories and specific subcategories, enabling multi-level analysis spanning minor administrative issues to major safety concerns.

### Regulatory explainability

The methodology supports regulatory transparency through: (1) statistical traceability with interpretable probability values, (2) topic provenance linking merged topics to original narratives, (3) human-in-the-loop design with manual quality control, and (4) documented model parameters supporting reproducibility. This aligns with FDA-EMA guidance on AI/ML emphasizing transparency and human oversight [18].

### Ethical considerations

Key considerations include:

1. **Fairness**: Sites flagged for investigation, not punitive action.
2. **Transparency**: Informing investigators about monitoring methodologies per ICH E6(R3).
3. **Bias mitigation**: Monitoring for systematic biases.
4. **Human oversight**: Final decisions remain with qualified personnel.

### Clinical and regulatory implications

The methodology’s ability to identify both low and high presence of PDs has important implications for regulatory compliance and data integrity [19]. Low presence can mask serious operational issues and compromise participant safety, while high may reflect skewed results related to safety, inadequate training, inconsistent interpretation of protocol requirements, or inefficient processes [20]. By flagging outliers and contextualizing their deviation content, sponsors can deploy targeted corrective and preventive actions (CAPA), reinforce training, and calibrate monitoring strategies to align with risk-based monitoring and quality-by-design principles [21].

Moreover, the approach facilitates early detection of investigator site- or region-specific trends (e.g., sample collection timing deviations), enabling proactive interventions before issues escalate. This supports regulatory expectations for continuous quality management, strengthens audit preparedness, and may reduce the likelihood of critical findings.

### Handling study-design variations

The bootstrap approach accounts for study-specific characteristics by generating expected distributions from within-study data. Each study serves as its own reference population, making the method robust to protocol complexity and visit schedule differences.

Misclassification Considerations: The methodology addresses potential misclassification of well-performing sites through: (1) topic analysis integration cross-referencing flagged sites with deviation topic distributions, (2) contextual investigation by Clinical Research Associates (CRA) and auditors, and (3) FDR-controlled significance thresholds reducing false positives.

Important Clarification: The bootstrap-based outlier detection is a signal detection approach, not a predictive model. Flagged sites represent statistical signals warranting investigation, not definitive compliance determinations, aligning with risk-based monitoring principles directing oversight toward sites most likely to benefit from scrutiny. Future enhancements could incorporate site-level covariates (investigator experience, monitoring frequency) as adjustment factors.

### Operational impact and value

In practice, the combined methodology demonstrates measurable benefits:

- **Trend identification**: Emerging deviation patterns missed by routine monitoring guide timely responses. Enhanced compliance: Focused remediation at flagged sites improves protocol adherence and reporting timeliness. Training optimization: Topic analysis pinpoints thematic gaps (e.g., informed consent documentation ), potentially enabling targeted training.
- **Resource allocation**: Risk profiling concentrates oversight (e.g.,CRA attention, Source Data Verification (SDV) focus) on high-risk sites, supporting efficiency without sacrificing quality.

### Scalability and future directions

Future developments include real-time monitoring, electronic data capture (EDC) integration, and expansion to other quality metrics [22]. Practical deployment requires: (1) standardized data pipelines for EDC/CTMS (Clinical trial management systems) integration; (2) streaming architecture for real-time monitoring; (3) governance frameworks for AI-assisted decision-making; (4) staff training on output interpretation; and (5) computer system validation depending on regulatory jurisdiction.

Despite the lack of gold-standard annotations, classification performance was assessed using an independent LLM-as-judge evaluation as described in Results, complemented by clinical operations expert review of category coherence and sensitivity analysis of clustering parameters. Observed limitations include potential underrepresentation of rare deviation types and variable performance on non-standard narrative entries. Full systematic bias assessment across demographic and geographic factors is planned for future research.

This integrated monitoring paradigm advances the industry toward proactive, system-aware clinical operations. Combining statistical rigor with AI-powered topic synthesis enables nuanced understanding of site behavior and trial quality dynamics, particularly for complex, geographically distributed trials where manual review is impractical. As AI models improve, topic libraries will help sponsors standardize best practices while benefiting participant safety, data integrity, and drug development efficiency. Real-world application demonstrates utility in improving trial oversight, proactive risk management, and regulatory compliance. By unveiling systematic issues not apparent through traditional methods, the approach supports targeted interventions and measurable gains in protocol adherence. Continued evolution toward real-time implementation will further strengthen its role in modern, risk-based clinical trial management.

### Limitations

The methodology requires sufficient historical data, limiting applicability to early-phase trials. Variability in narrative quality affects AI-powered analysis, necessitating human-in-the-loop quality control. The implementation has not been systematically validated against human-annotated gold-standard datasets. Future work should include: (1) expert-annotated datasets, (2) inter-rater reliability assessment and (3) precision/recall calculations. Site-level cultural and operational differences may influence reporting behavior in ways not fully captured by current models. Finally, broader integration with operational systems and governance frameworks is needed to enable real-time deployment and support regulatory acceptance [22–23].

## Data Availability

The operational data used in this article are not publicly available.

## Abbreviations

CAPA: Corrective and preventive actions
CRA: Clinical research associate
CTMS: Clinical trial management systems
CV: Coefficient of variation
EDC: Electronic data capture
EMA: European medicines agency
FDA: Food and drug administration
FDR: False discovery rate
GenAI: Generative artificial intelligence
ICH: International council for harmonisation
LLM: Large language model
OSTIC: Office of scientific and technical information clearance
PD: Protocol deviation
RCT: Randomized controlled trial
SDV: Source data verification
